# Tumoral chromoblastomycosis: a rare manifestation with typical complementary exams*

**DOI:** 10.1590/abd1806-4841.20154490

**Published:** 2015

**Authors:** John Verrinder Veasey, Beatriz de Abreu Ribeiro Machado, Rute Facchini Lellis, Laura Hitomi Muramatu, Clarisse Zaitz

**Affiliations:** 1 Irmandade Santa Casa de Misericórdia de São Paulo - São Paulo (SP), Brazil

**Keywords:** Chromoblastomycosis, Diagnosis, Diagnostic tests, routine, Histology, Mycology, Microscopy, Physical examination

## Abstract

Chromoblastomycosis is a chronic subcutaneous fungal infection caused by traumatic implantation of dematiaceous fungi in the skin. The clinical presentation is usually a verrucous plaque lesion and the diagnosis is confirmed by the visualization of muriform bodies at direct examination or at the histologic study. This report describes a rare case of tumoral chromoblastomycosis confirmed by histologic study and whose agent was identified by culture and micromorphology.

Man, 77 years old, institutionalized for psychiatric history. He was taken to dermatological evaluation due to a painless tumor in his left knee that was presenting a progressive growth for a year, and the patient denied previous trauma. Physical examination revealed a tumor in his left knee, pinkish in color, measuring 3 centimeters in major axis (Figure 1). In the direct mycological examination with 20% KOH, the research for fungal structures was negative. A biopsy of the lesion was performed and anatomopathological studies showed epidermis with moderate irregular acanthosis and dermis presenting lymphohistiocytic infiltrate with granulomatous outline, and some giant cells containing muriform bodies (Figure 2). Culture of fragment of the material in Sabouraud agar showed growth of nonspecific dematiaceous cottony colony and microcultivation showed *Cladosporium* type fructification, confirming the hypothesis of chromoblastomycosis by agent of the complex *Fonsecacea pedrosoi *(Figures 3 and 4).

**Figure 1:**
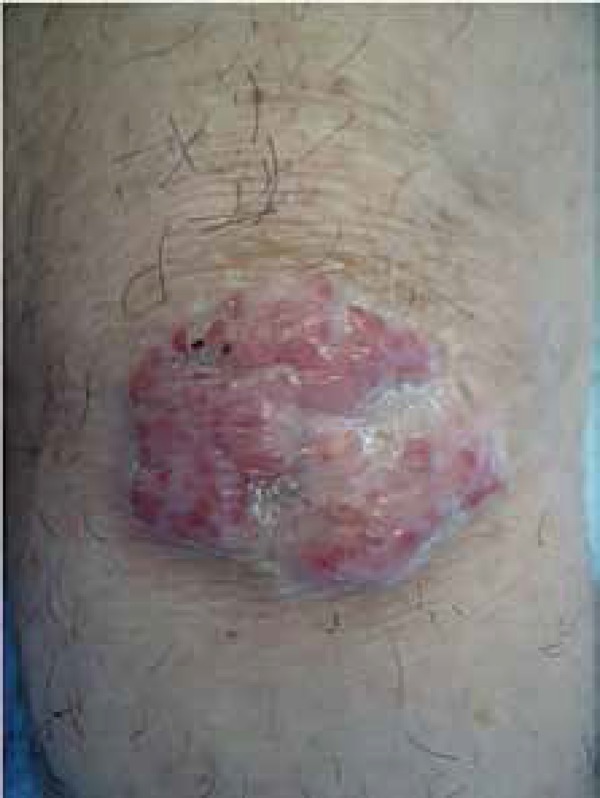
Tumor in left knee, pinkish in color, measuring 3 cm in major axis

**Figure 2:**
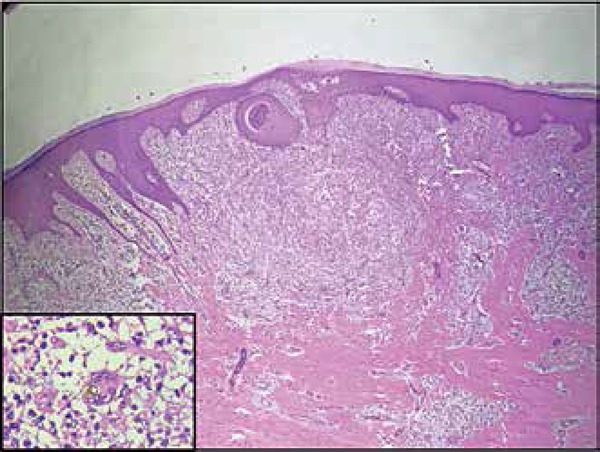
Histologic examination (x100): epidermis with moderate irregular acanthosis, dermis with lymphohistiocytic infiltrate presenting granulomatous outline and some giant cells containing muriform bodies (detail, x 400)

**Figure 3:**
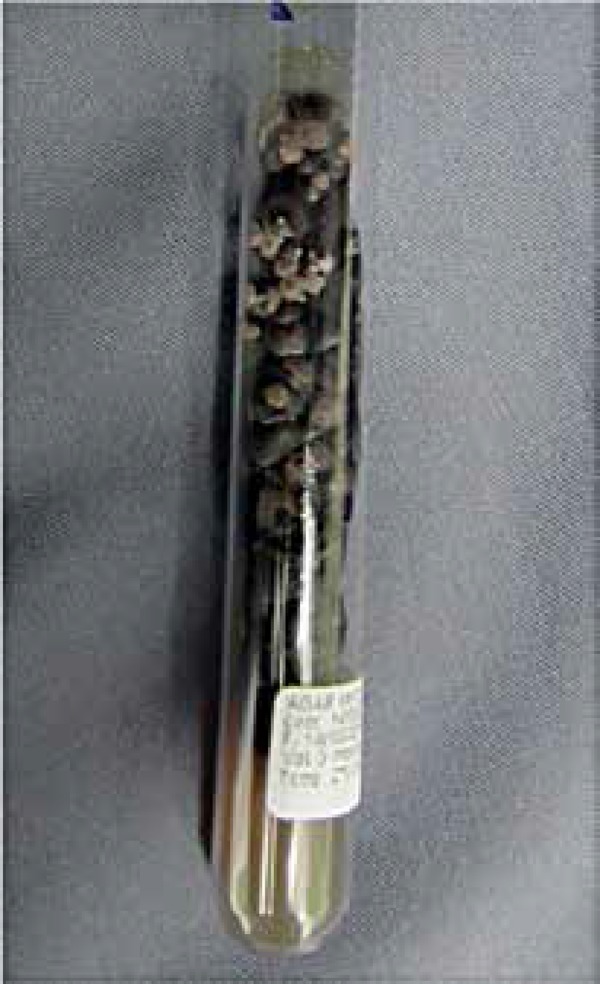
Culture in Sabouraud agar presenting cottony dematiaceous colony

**Figure 4:**
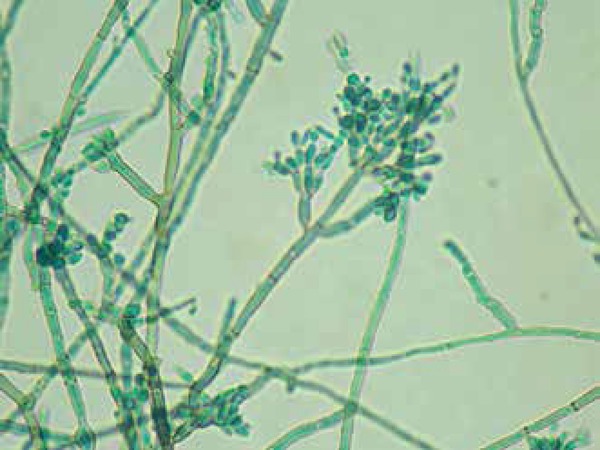
Microcultivation with septated dematiaceous hyphae and *Cladosporium* type fructification

## DISCUSSION

The described clinical presentation is the rarest of the chromoblastomycosis condition, however its location in the lower limbs and male involvement remains similar to other cases.^1-3^ The negative mycological direct examination can be interpreted consistently with the clinical form, since the epidermal lesion presented full, making it difficult to collect material containing some fungal structure; at the same time, recent publications on the subject highlight that in about 6% of cases the examination may be negative.^2^ Despite the peculiarities of the case, the identified agent is from the complex *Fonsecacea pedrosoi*, the main agent of chromoblastomycosis in our country.^1-3^
